# Serum Cortisol and Interleukin-6 as Key Biomarkers for a Diagnostic Algorithm of Combat-Related PTSD

**DOI:** 10.3390/brainsci15121319

**Published:** 2025-12-10

**Authors:** Yana Zorkina, Alexander Berdalin, Olga Abramova, Aleksandr Reznik, Valeriya Ushakova, Vladimir Mukhin, Daria Riabinina, Alina Khamidova, Olga Pavlova, Konstantin Pavlov, Elizaveta Golubeva, Angelina Zeltser, Georgy Kostyuk, Anna Morozova

**Affiliations:** 1 Mental-Health Clinic No. 1 Named After N.A. Alekseev, Zagorodnoe Highway 2, 115191 Moscow, Russia; alex_berdalin@mail.ru (A.B.); a.m.reznik1969@yandex.ru (A.R.); khamidovaar1@zdrav.mos.ru (A.K.);; 2 Department of Basic and Applied Neurobiology, V. Serbsky Federal Medical Research Centre of Psychiatry and Narcology, Kropotkinsky per. 23, 119034 Moscow, Russia; 3 Mental-Health Clinic No. 4 Named After P.B. Gannushkin, 107076 Moscow, Russia; 4 Moscow Regional Mental-Health Hospital No. 5, 141370 Khotkovo, Russia; 5 Department of Psychiatry, Federal State Budgetary Educational Institution of Higher Education “Russian Biotechnology University”, Volokolamskoye Highway 11, 125080 Moscow, Russia; 6 Faculty of Psychology, M.V. Lomonosov Moscow State University, 119991 Moscow, Russia

**Keywords:** PTSD, biomarker, diagnostics, cortisol, IL-6, neuroinflammation, HPA axis, combat stress

## Abstract

Background: Post-traumatic stress disorder (PTSD) is a severe psychiatric condition prevalent among combat veterans. Its diagnosis is challenging due to the heterogeneity of clinical presentations and the complex interplay of pathogenic factors. Objective: This study aimed to develop and validate a diagnostic algorithm for combat-related PTSD by integrating clinical data with a panel of biological markers associated with blood–brain barrier disruption (anti-GFAP and anti-NSE antibodies), HPA axis dysfunction (cortisol), and neuroinflammation (IL-6, IL-8). Methods: A total of 721 male participants were enrolled: 434 veterans with PTSD (F43.1), 147 combat veterans without PTSD, and 140 non-combat military controls. All participants underwent clinical and psychometric assessment (Likert scale, HADS). Serum levels of biomarkers were measured using ELISA. Statistical analysis included non-parametric tests, correlation analysis, and binary logistic regression with Wald’s method to build a predictive model. Results: The binary logistic regression model identified cortisol and IL-6 as the most significant predictors of PTSD. The final algorithm, based on a cortisol level below 199.8 nmol/L and an IL-6 level above 0.002438 pg/mL, correctly classified 78% of patients (AUC = 0.724, 95% CI [0.669, 0.779]). Furthermore, levels of IL-4, IL-8, and cortisol positively correlated with the severity of combat stress factors, independent of physical injuries. Conclusions: We developed a novel diagnostic algorithm for combat-related PTSD based on cortisol and IL-6 levels, demonstrating high accuracy. The correlation between neuroinflammatory markers and the severity of combat exposure suggests their role as primary indicators of stress response, highlighting their utility for early risk identification and targeted interventions.

## 1. Introduction

Post-traumatic stress disorder (PTSD) is a severe mental condition that results from exposure to extreme traumatic events, such as participation in combat. The prevalence of PTSD among veterans varies from 10% to 30%, highlighting its significance as a public health issue [1].

The difficulty of diagnosing mental disorders in combat participants is explained by the following reasons. The diagnosis of combat-related mental disorders is complex due to several interconnected factors. First, military personnel represent a heterogeneous group with varying pre-existing mental health, including chronic conditions [2]. Stress-related disorders arise from a confluence of pre-deployment vulnerabilities and war-specific pathogenic factors, such as psychological trauma, physical injuries (including concussion), and severe exhaustion. Post-deployment challenges that contradict expectations can further exacerbate these conditions [3,4,5,6]. Clinically, this can manifest not only as classic PTSD but also as complex forms featuring emotional instability, personality changes, severe depression, and even psychotic or dissociative symptoms [6,7,8]. Crucially, comorbid traumatic brain injury (TBI) is a major etiological factor that can cause neurological sequelae, lower stress resilience, and complicate differential diagnosis by blurring the lines between psychogenic and organic origins [9,10,11,12]. Finally, PTSD itself presents with a wide spectrum of comorbid disorders that vary in structure, severity, and functional impact [2,6,7]. In summary, combat-related mental pathology, including PTSD, arises in individuals with varying predispositions under the influence of a multifactorial pathogenic milieu. These factors most often act concurrently and over prolonged periods, resulting in mixed psychopathological presentations and diverse clinical trajectories.

In this regard, in the real clinical practice of providing assistance to survivors of combat mental trauma, there are significant difficulties with differential diagnosis, identifying the primary debilitating symptom cluster, and establishing the optimal target of therapy at each stage of patient management and predicting treatment outcomes.

Our study’s focus on combat-related PTSD is justified by its distinct clinical presentation and unique etiological profile. While the core symptoms of re-experiencing, avoidance, and hyperarousal are universal, the combat phenotype is frequently characterized by greater severity of irritability, explosive anger, and comorbid somatic complaints [2]. Combination of chronic psychological trauma and physical injury creates a specific pathogenetic pathway that distinguishes combat PTSD from other forms of the disorder and necessitates tailored diagnostic approaches.

One of the promising approaches is the identification of predictive biological markers associated with various psychopathological manifestations of combat stress disorders. In recent years, substantial evidence has implicated several core pathophysiological pathways in PTSD. In PTSD, there is a disruption in the functioning of various biological processes that are interconnected [13]. Pathogenetic mechanisms may have different meanings in different people. Consequently, it is not enough to use one or more markers that reflect only one pathogenetic process for an objective diagnosis. Critically, the biological disruptions that confer vulnerability to PTSD often persist in the chronic phase of the illness, making them relevant targets for diagnostic algorithms.

Dysregulation of the hypothalamic–pituitary–adrenal (HPA) axis is a cornerstone of the stress response, and individual variations in its function may constitute a key endophenotypic predisposition to stress-related mental disorders Disruption of the hypothalamic–pituitary–adrenal HPA axis is a key point of stress exposure, and biological endophenotypes of HPA may be a predisposition factor for the development of stress-related mental disorders [14]. Circulating cortisol levels measured shortly after trauma exposure have been established as a significant predictor of subsequent psychopathology [15,16]. Beyond peripheral cortisol levels, other promising markers of HPA axis regulation include epigenetic modifications, particularly the methylation status of genes critical to glucocorticoid signaling such as FKBP5 and NR3C1 [17].

A second major category of predictive biomarkers involves inflammatory mediators. Pro-inflammatory cytokines (e.g., IL-6, IL-1β, TNF-α) and acute-phase proteins like C-reactive protein, which elevate following traumatic exposure, have been consistently linked to the development of PTSD, underscoring the role of systemic inflammation in its etiology [18]. This peripheral inflammatory response is often paralleled by neuroinflammation, which is closely associated with impaired blood–brain barrier (BBB) integrity [19]. Given that combat-related PTSD is frequently comorbid with concussions and traumatic brain injuries—conditions known to exacerbate PTSD severity—the relevance of BBB disruption is particularly salient [20]. Indeed, BBB compromise has been implicated in numerous stress-related psychiatric disorders [21] and is mechanistically intertwined with the central nervous system’s inflammatory response [19]. These processes form a tightly linked pathogenic cycle, making it difficult to delineate a primary initiating event [22].

Another promising category of predictive biomarkers comprises genetic markers. Their principal theoretical advantage lies in their stability, being present from birth and unaffected by the disease state, thus offering insight into innate vulnerability. However, while genetic studies, particularly genome-wide association studies (GWAS), aim to uncover these heritable risk loci, the findings have been largely inconsistent and lack replication, underscoring the highly polygenic and heterogeneous nature of PTSD. Therefore, their current utility in widespread clinical practice remains limited [23].

The ultimate goal of identifying robust predictive biomarkers is to enable early detection of high-risk individuals, thereby facilitating timely preventive interventions and potentially mitigating the onset of the disorder [24].

Despite numerous investigations into potential biomarkers, so far no attempts have been made to identify the most significant ones for subsequent diagnosis. To address this gap, we strategically prioritized a panel of biomarkers selected for their dual relevance to the core pathophysiology of combat-related PTSD and their practical measurability in routine clinical settings.

Consequently, this study aimed to develop a practical diagnostic algorithm based on biomarkers that fulfill two key criteria: (1) they reflect the core pathophysiological pathways in combat-related PTSD (HPA axis dysfunction, neuroinflammation, and BBB disruption associated with frequent TBIs), and (2) they can be routinely quantified using simple, widely available, and cost-effective ELISA techniques. This represents a deliberate compromise between pathophysiological comprehensiveness and clinical practicality, chosen to maximize the algorithm’s potential for widespread adoption and implementation.

Thus, the specific objective of this work was to create and validate a diagnostic algorithm for combat-related PTSD that integrates clinical data with a panel of serum biomarkers, including markers of BBB integrity (antibody levels to glial fibrillary acidic protein (GFAP) and neuron-specific enolase (NSE)), HPA axis dysfunction (cortisol), and neuroinflammation (levels of interleukin-6 (IL-6) and interleukin-8 (IL-8)).

## 2. Materials and Methods

Study Participants

A total of 721 male participants were enrolled in this study and allocated into three groups:

- PTSD Group (*n* = 434). This group comprised military veterans diagnosed with PTSD (F43.1) who were hospitalized to the Psychiatric Clinical Hospital No. 1 named after N.A. Alekseev in an emergency or planned order;
- Combat-exposed Control Group (*n* = 147). This group consisted of veterans with a history of combat exposure but no clinical diagnosis of PTSD. They were recruited from the neurological department of Veterans Hospital No. 3;
- The control group (*n* = 140) consisted of reserve military personnel who did not participate in combat and did not have a psychiatric diagnosis.

Diagnoses for the PTSD and noPTSD groups were confirmed by the attending physician in accordance with International Classification of Diseases (ICD-10) criteria. For all participants, inclusion in the study and initial assessment were completed within two weeks of hospital admission.

The inclusion criteria for the two veteran groups (PTSD and combat-exposed controls) were: direct participation in combat operations with exposure to traumatic events; male gender; and provision of written informed consent for both treatment and the use of personal data for research purposes.

Psychometric scales

All data were collected using standardized procedures, including clinical and anamnestic analysis, psychometric testing, and structured interviews. The following variables were recorded for each participant: age, time since last combat exposure (in weeks), total duration of combat stress (cumulative time spent in a combat zone), and an overall severity index of combat-related traumatic factors.

The presence and severity of PTSD symptoms were assessed using a five-point Likert scale designed to align with ICD-10 diagnostic criteria. The rated symptoms included depression, personality changes, avoidance, irritability, intrusive thoughts, and sleep disturbances.

Hospital Anxiety and Depression Scale (HADS) was employed to screen for generalized symptoms of anxiety and depression. HADS is a 14-item self-report screening instrument divided into two subscales: the HADS-A (7 items) for generalized anxiety symptoms and the HADS-D (7 items) for the affective dimension of depression. HADS includes specific measures that determine general anxiety, including tension, anxiety, fear, panic, difficulty relaxing and restlessness [25].

Serum samples collection

Blood parameters were measured in serum. Blood samples for analysis were collected from the cubital vein in the fasting state, typically before 9 a.m. Serum was isolated immediately following blood collection via centrifugation at 3000 rpm for 10 min at 4 °C, and then stored at –80 °C until analysis.

Determination of the concentration of neurospecific antibodies to Neuron-Specific Enolase (NSE) and Glial fibrillary acidic protein (GFAP)

Recombinant GFAP and NSE proteins, previously obtained [26,27], were used as antigens for analysis. Recombinant proteins (GFAP, NSE) were immobilized at the bottom of the wells of a 96-well tablet for 12 h at a temperature of +4 °C in 0.1 M NAHCO_3_, pH 9.2. After that, the solution was removed and the tablet was washed with a washing buffer solution (phosphate-buffered saline (PBS), 0.05% Twin-20, pH 7.4) using the WellWash automatic mixer (ThermoScientific, Waltham, MA, USA). Then, non-specific binding was blocked by incubation with 3% bovine serum albumin on PBS for 1 h at room temperature. After that, the tablet was washed and 100 µL of patient serum samples were added (dilution 1:50, PBS). Incubation lasted 1.5 h at room temperature on a shaker incubator (500 rpm). Then the tablet was washed with a washing buffer solution and secondary antibodies were added—antibodies to human immunoglobulins conjugated with biotin (at a dilution of 1:40,000, ThermoFisher, Waltham, WA, USA) for 1 h at room temperature (500 rpm). After washing, streptavidin conjugate with peroxidase was added (at a dilution of 1:5000, ThermoFisher, USA) for 30 min at room temperature (500 rpm). The enzymatic activity of peroxidase was demonstrated using a solution of the chromogenic substrate tetramethylabenzidine (Amresco, Framingham, MA, USA) for 10 min. Then the enzymatic reaction was stopped by adding a solution of 1 M H_2_SO_4_. The results were read using a Varioscan LUX photometer (ThermoScientific, USA) at a wavelength of 450 nm.

Determination of the IL-6,8 and cortisol concentration

The concentration of cortisol, IL-6 and IL-8 was assessed by enzyme immunoassay (ELISA) using commercial ELISA kits manufactured by JSC VECTO-BEST, Russia («Interleukin-6-ELISA-BEST», «Interleukin-8-ELISA-BEST», «Cortisol-ELISA-BEST»). The concentration assessment was carried out in accordance with the manufacturer’s protocol.

Statistical Processing

Analysis was performed using jamovi 2.6.26 and RStudio software 4.5.2. Upon receiving the data, its distribution was assessed for normality using the Shapiro–Wilk test. This assessment determined the appropriate criteria for statistical comparison: parametric or non-parametric. The analysis showed that all data in the study were not normally distributed. Non-parametric analysis was performed using the Kruskal–Wallis test and subsequent multiple comparison tests (Dwass–Steel–Critchlow–Fligner), the data were presented as Median (Q1, Q3). Differences were considered statistically significant at *p* < 0.05. Comparison of groups by parameters Injury, Pain syndrome was carried out using the chi-square criterion.

Pearson correlation analysis was used to assess the relationship between quantitative variables. FDR multiplicity correction by Benjamini–Hochberg procedure were applied to correlation analysis results.

To build a predictive model of PTSD based on generalized data, binary logistic regression with a sequential selection of predictors using the Wald method was applied. To select a cumulative predictive model, the sample was randomly divided into a training and a test sample in a ratio of 33%/66%.

The dependent variable was a binary variable reflecting the presence or absence of PTSD in all the examined patients. The predictors were variables that differ between the study groups. Before model building multicollinearity diagnostics were performed (VIF for final model were 1.7). Nagelkerke pseudo-R-squared, and the significance of the Hosmer-Lemeshev test were used as model quality metrics.

ROC-analysis were used to check final regression model quality and to determine cut-off points for individual significant predictors. In both cases comparison groups were—PTSD and no-PTSD. For model quality predicted probability were dependent variable. Cut-off points was selected using Youden index calculation.

## 3. Results

Assessment of the level of stress and clinical information of participants

Participant age was compared across all three groups. The Kruskal–Wallis test revealed a statistically significant difference in age (χ^2^ = 41.8, *p* < 0.001). Post hoc analysis showed that the control group was significantly older than both the PTSD group and the noPTSD group (*p* < 0.001). The average age in the groups was: the control group-43 years (34; 52), the PTSD group—34 years (28; 42), the noPTSD group—35.5 years (29.3; 42.7).

The data on the severity of stress and clinical characteristics of patients from two experimental groups, PTSD and noPTSD, were analyzed. While the two groups did not differ in the total duration of combat exposure or the time since leaving the conflict zone, significant differences were observed in all psychometric measures (see Table 1). Individuals in the PTSD group exhibited significantly higher overall levels of PTSD symptoms, as well as increased HADS scores (for depression and anxiety) and the Likert scale (for depression, personality traits, avoidance, irritability, and intrusive thoughts) compared to those in the noPTSD group. There were no differences between the two groups in terms of sleep disorders as measured by the Likert scale (see Table 1).

Assessment of the concentration of biochemical markers in blood

Statistically significant differences were found between the groups in all five parameters: antibodies to GFAP, antibodies to NSE, cortisol, IL-6, IL-8 (Table 2).

**Figure 1:**
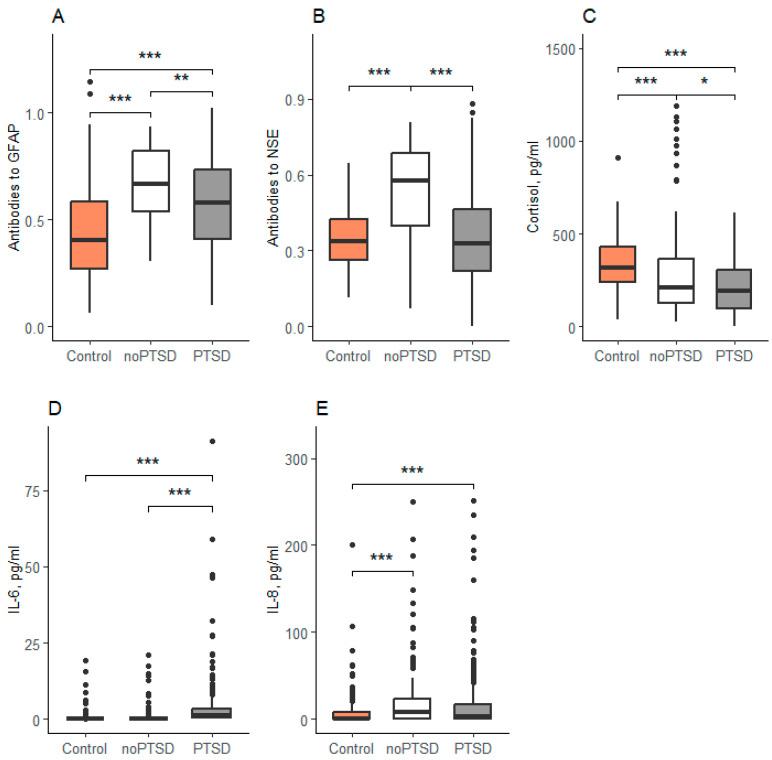
Concentration of biochemical parameters in serum. Data are expressed as Med (Q1; Q3) in boxplots. (**A**)—Concentration of antibodies to GFAP, (**B**)—Concentration of antibidies to NSE, (**C**)—Concentration of cortisol, (**D**)—Concentration of IL-6, (**E**)—Concentration of IL-8. Black horizontal lines—medians; Black points—outliers; *—<0.05, **—<0.01, ***—<0.001; Control-reserve military personnel who did not participate in combat, noPTSD is a group of combat veterans who have not developed PTSD, PTSD is a group of combat veterans who have developed PTSD.

Post hoc analysis revealed significant intergroup differences across all measured biomarkers (Figure 1). Anti-GFAP antibody levels differed significantly between all three groups. The control group demonstrated lower concentrations than both the PTSD (*p* < 0.001) and noPTSD (*p* < 0.001) groups. Furthermore, the PTSD group exhibited lower GFAP antibody levels than the noPTSD group (*p* = 0.008).

In contrast, anti-NSE antibody levels were significantly elevated in the noPTSD group compared to both the control (*p* < 0.001) and PTSD (*p* < 0.001) groups, with no significant difference observed between the latter two (*p* = 0.78). Cortisol levels were highest in the control group and were significantly reduced in both the PTSD and noPTSD groups (both *p* < 0.001). Cortisol was also significantly lower in the PTSD group compared to the noPTSD group (*p* = 0.03). Similarly, IL-6 concentrations were significantly elevated in the PTSD group relative to both the control and noPTSD groups (both *p* < 0.001; Figure 1D). Finally, IL-8 levels were higher in both combat-exposed groups (PTSD and noPTSD) compared to the non-combat controls, with no significant difference between the two veteran groups (Figure 1E).

Development of a diagnostic algorithm model.

The outcome variable was a binary indicator of the presence or absence of PTSD among all patients studied. Predictors were selected based on differences between the study groups, and the final model was developed using a training sample that was randomly selected from the original dataset. The training sample was split into a 33%/66% training/testing ratio.

During the sequential selection of Wald predictors within the framework of binary logistic regression, Cortisol (*p* < 0.0005, OR = 0.995 95% CI [0.992, 0.998]) and IL-6 (*p* = 0.015; OR = 1.346 95% CI [1.061, 1.708]) were selected from potential predictors (Table 3). The model allowed correct classification of 78% of patients. The total model turned out to be significant (Chi-squared = 27.794, *p* < 0.0005), Nagelkerke pseudo-R-squared = 0.270, and the significance of the Hosmer-Lemeshev test *p* = 0.399.

The observed statistical power for the model in the training sample was 77%. Notably, the PTSD and non-PTSD groups did not differ significantly in age or time since combat deployment. The forced inclusion of these non-significant covariates for adjustment was found to reduce both the model’s statistical power and its overall classificatory performance.

The quality of the classification model.

**Figure 2:**
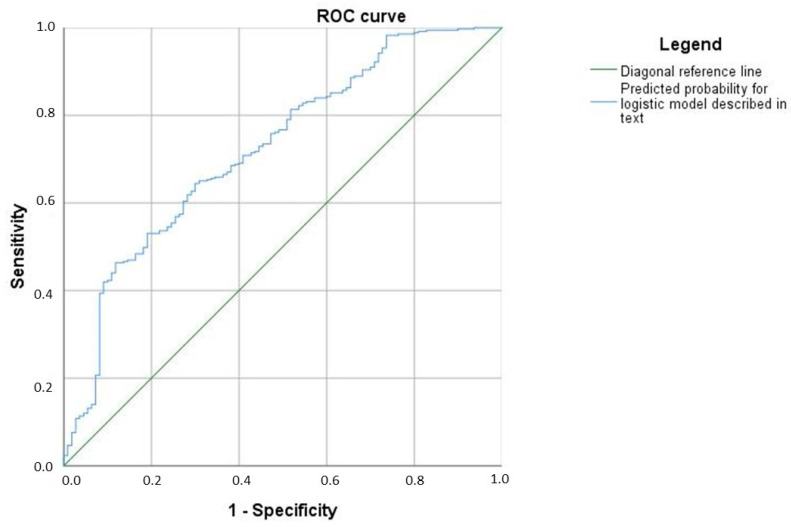
A model of the diagnostic algorithm.

According to the ROC analysis, the area under the curve for the predicted probabilities is 0.724 (95% CI [0.669, 0.779]), Figure 2.

For IL-6, the critical value was 0.002438, i.e., if we assume that all patients with an interleukin concentration greater than the above value have PTSD, then the sensitivity of this judgment will be 79.1%, and the specificity will be 79.3%.

For cortisol, the cut–off point was 199.8. A cortisol concentration below this value predicted PTSD with a sensitivity and specificity of 53.2%.

Therefore, a combined criterion of IL-6 > 0.002438 pg/mL and cortisol < 199.8 nmol/L can be indicative of PTSD.

Dependence of the concentration of biomarkers on the severity of combat factors

**Figure 3:**
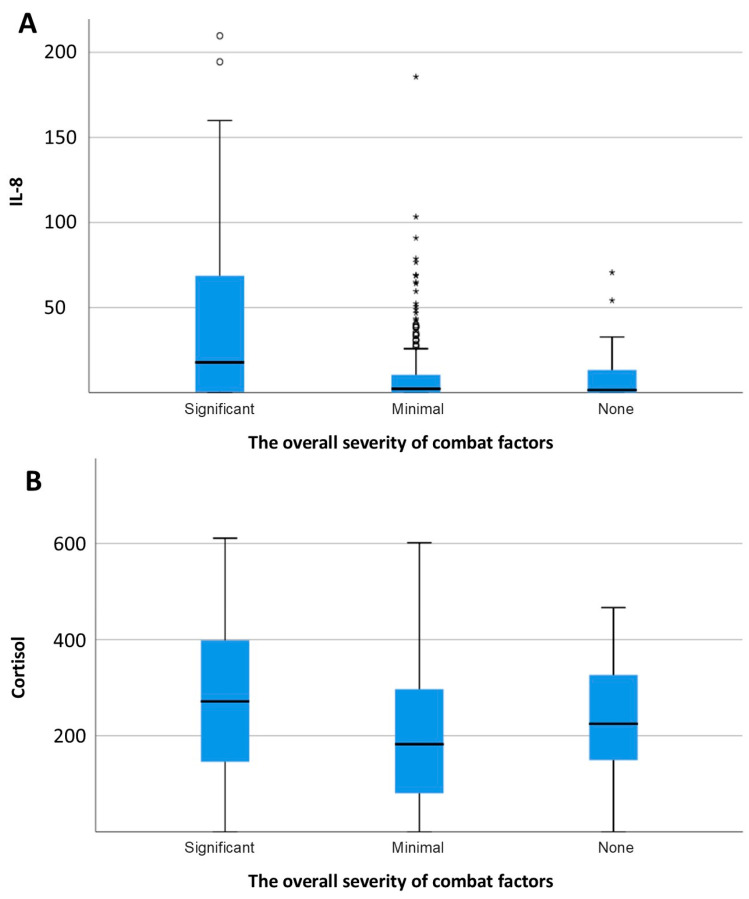
Concentration of IL-8 (**A**) and cortisol (**B**) depending on the overall severity of combat factors. * *p* <0.05.

**Figure 4:**
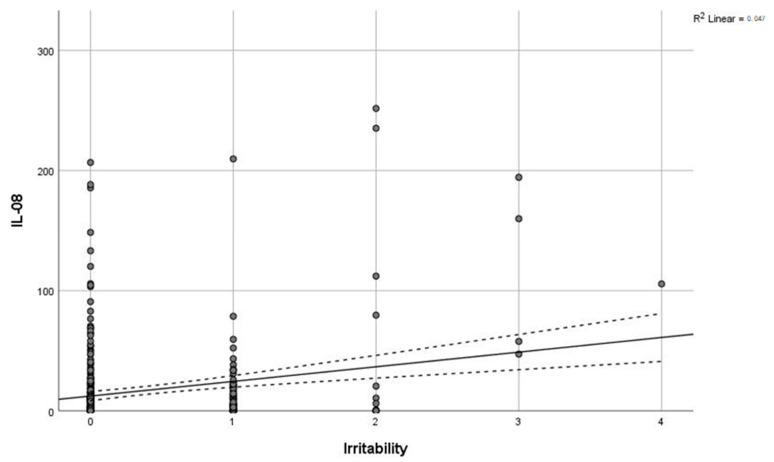
Correlation of IL-8 with irritability. Grey line—regression line, dashed line—95% confidence interval.

Levels of IL-8 (F = 31, *p* < 0.0005) and cortisol (F = 5.3, *p* = 0.005) demonstrated a significant positive association with the severity of combat exposure (Figure 3). Furthermore, a significant positive correlation was observed between excitability scores and IL-8 levels (r = 0.216, *p* < 0.001; Figure 4). In contrast, concentrations of IL-6 and IL-8 were not significantly different between combatants with and without physical injury or pain syndrome.

## 4. Discussion

The present study aimed to develop a blood-based predictive model for PTSD utilizing widely available and cost-effective biomarkers. During the development of the diagnostic algorithm, we focused on biomarkers commonly associated with stress-related mental disorders. Our analysis revealed statistically significant differences in the levels of several biomarkers—namely, antibodies to GFAP, antibodies to NSE, cortisol, and IL-6—between patients with PTSD and those without. Critically, these specific biomarkers are of particular interest because they distinguish individuals who developed PTSD from those who did not, despite shared exposure to the severe traumatic context of combat. This differential expression highlights their potential utility in identifying the distinct pathophysiological responses that underlie PTSD susceptibility versus resilience within a homogeneously traumatized cohort. An additional biomarker, IL-8, also demonstrated a significant deviation from control levels. We subsequently demonstrated that these parameters could be integrated into a functional model for PTSD identification.

Our findings contribute to the growing effort to define a blood-based diagnostic signature for PTSD. For instance, one multi-omics study identified a panel of 28 markers based on their efficacy and ability to track phenotypic changes over time, reporting robust results (AUC = 0.80, accuracy 81%, sensitivity 85%, and specificity 77%) [28]. While such comprehensive approaches are valuable, their complexity and cost may limit widespread clinical implementation. In contrast, the primary objective of our work was to develop a more compact, clinically practical, and economically feasible alternative without compromising diagnostic utility.

The cornerstone of our final model was reduced blood cortisol levels. A substantial body of evidence indicates that PTSD is characterized by hypocortisolemia. A meta-analysis by Morris et al. confirmed significantly lower cortisol levels in individuals with PTSD [29]. This phenomenon is attributed to a physiological mechanism involving HPA axis dysregulation: individuals with PTSD exhibit hyperactivity of corticotropin-releasing hormone (CRH) coupled with subsequent hypersensitivity of glucocorticoid receptors. This leads to enhanced negative feedback inhibition of cortisol and increased CRH release [30]. Therefore, the inclusion of cortisol in a predictive model for PTSD is well-justified, and its measurement shortly after trauma exposure represents a promising tool for risk stratification.

HPA axis dysfunction is intrinsically linked to the development of neuroinflammatory processes [31]. Reduced glucocorticoid activity in chronic PTSD, combined with decreased parasympathetic and increased sympathetic nervous system tone, creates a permissive environment for a chronic pro-inflammatory state [32]. Numerous studies have reported alterations in peripheral pro-inflammatory biomarkers in individuals with PTSD [33,34]. A meta-analysis by Passos et al. (2015) further substantiated that IFN-γ, interleukin-1β (IL-1β), IL-6, and tumor necrosis factor-α (TNF-α) are the most consistently elevated pro-inflammatory cytokines in the blood of PTSD patients compared to healthy controls [35]. The results of our study, which identified a significant elevation of the pro-inflammatory cytokines IL-6 and IL-8 in the PTSD group, are in direct alignment with these established findings.

The neuroinflammatory response is closely associated with dysfunction of the BBB. The BBB maintains homeostasis within the nervous system by regulating the interaction between endothelial cells, astrocytes, pericytes, and microglia [36]. Activated glial cells can increase BBB permeability by altering tight junctions and activating adhesion molecules. This, in turn, promotes the migration of immune cells into brain tissues, leading to a vicious cycle of heightened inflammation and further brain damage [19]. Reactive astrocytes exhibit an A1 phenotype, characterized by increased GFAP expression [37]. The presence of antibodies to GFAP signifies not only BBB disruption but also that this disruption was sufficient to trigger an autoimmune response [38]. This allows for the detection of BBB impairment even some time after an acute injury. The presence of antibodies reflects longer-term or more severe damage—sufficient for the immune system to mount an adaptive response, as has been demonstrated in traumatic brain injury [38].

In contrast to antibodies, the level of the GFAP itself returns to baseline after the acute injury subsides and GFAP is cleared from the bloodstream, primarily serving as a short-term indicator of BBB damage [39]. In our study, we detected antibodies against GFAP in the blood, which are formed as a consequence of an immune response to brain antigens that were previously shielded from the immune system by the intact BBB. Indirectly, the elevation of these antibodies indicates not only impaired BBB permeability but also the development of an astrocytic response at the site of damaged nervous tissue. In our investigation, anti-GFAP antibody levels were elevated in the groups exposed to traumatic stress, while they were reduced in the PTSD group. This observation can be attributed to the fact that antibody production increases not due to the mental trauma per se, but rather as a response to the traumatic events themselves. Reactive astrogliosis, a hallmark of this process, may also contribute to this finding [40].

The presence of anti-NSE antibodies provides further evidence of BBB compromise. In our study, anti-NSE levels were elevated in all combat-exposed individuals compared to non-combat controls, but did not differ significantly between the PTSD and noPTSD groups. This pattern suggests that BBB dysfunction, as indicated by anti-NSE seropositivity, is a general consequence of severe trauma exposure rather than a specific marker of PTSD pathogenesis. To our knowledge, no prior studies have investigated antibodies against neurospecific proteins in the context of PTSD or psychological stress. We propose that this serological approach is particularly advantageous for psychiatric research, as the detection of the neurospecific proteins themselves (NSE, GFAP) in blood is transient and non-specific, typically associated with acute neurological damage [39], whereas antibodies provide a more stable, longer-term indicator of a prior immune challenge to the CNS.

Acute stress, alongside traumatic brain injury, constitutes a major driver of the neuroinflammatory process [41]. A key unresolved question is whether neuroinflammation is a primary event that contributes to the development of mental disorders or a secondary consequence of the psychopathology itself. To address this, we investigated the interrelationships between neuroinflammatory markers, including those not directly associated with physical injury, pain, or the specific severity of traumatic exposure.

Our analysis revealed no significant correlations between physical injury or pain syndrome and the levels of neuroinflammatory markers. However, a positive correlation was identified between IL-8 and cortisol concentrations and the severity of combat-related factors. This finding points to a direct link between the intensity of psychological trauma and the activation of specific neuroinflammatory and neuroendocrine pathways during military engagement. This aligns with meta-analytic evidence confirming that traumatic exposure itself can elevate cytokine levels [42]. Consequently, our data suggest that the neuroinflammatory processes observed in our cohort are likely primary phenomena triggered by the combat experience itself, rather than secondary developments ensuing from the clinical onset of a mental disorder.

The influence of TBI on both the diagnosis of PTSD and associated blood biomarker profiles constitutes a critical area for discussion. TBI is a major medical concern, associated with a high risk of severe long-term neurological sequelae, as well as chronic cognitive, behavioral, and psychiatric impairments, including neurodegenerative conditions [43]. In military contexts, TBI has become a predominant concern, often described as a “signature injury” due to the prevalence of blast exposure as a primary etiological mechanism. A robust association between TBI and PTSD has been established, with studies indicating that up to half of individuals with mild TBI meet diagnostic criteria for PTSD [44]. TBI is associated with disruption of the BBB, as evidenced by elevated serum levels of specific BBB integrity markers such as S100B, GFAP, and NSE [43]. This phenomenon was partially observed in our study through the alterations in anti-GFAP and anti-NSE antibodies, suggesting a potential BBB compromise in our patients with PTSD. While this compromise may be attributable to comorbid TBI, this hypothesis requires further dedicated investigation. The presence, severity, and chronicity of TBI can significantly confound peripheral biomarker measurements. In our cohort, a defined percentage of combat veterans reported a history of injury. Although the study groups were comparable in this regard, the potential confounding influence of this factor must be considered. A significant limitation of the present study is the inability to precisely account for the timing and severity of injuries due to methodological constraints. Consequently, the specific impact of TBI on the observed biomarker levels could not be accurately delineated. A systematic investigation of this relationship is an essential objective for future research.

The findings of this study hold significant promise for enhancing clinical practice by providing a biologically grounded, adjunctive tool for the objective assessment of combat-related PTSD, leveraging the combined measurement of serum cortisol (<199.8 nmol/L) and IL-6 (>0.002438 pg/mL), which are both routinely available and cost-effective assays, thereby facilitating potential integration into standard clinical workflows. While the model’s moderate accuracy (78%, AUC = 0.724) precludes its use as a standalone diagnostic, it serves as a valuable supplementary instrument for risk stratification and can aid in clarifying complex diagnostic presentations. Furthermore, the identified correlation between neuroinflammatory markers (IL-8) and the severity of combat exposure, independent of physical injury, suggests their utility as early screening biomarkers to identify at-risk individuals prior to the full manifestation of the disorder, enabling timely preventive interventions. The specific biomarker profile of hypocortisolemia and elevated pro-inflammatory cytokines offers a pathophysiological rationale for exploring targeted treatments, such as anti-inflammatory therapies or HPA-axis modulators. Finally, the discovery of elevated anti-GFAP antibodies following traumatic stress, even in the absence of PTSD, provides novel insights into the neuroautoimmune consequences of combat and underscores the potential for long-term neurological sequelae, warranting further investigation.

This study has several limitations that should be considered when interpreting the results. The assessment of PTSD relied on clinical diagnosis and a Likert scale rather than a gold-standard, structured clinical interview such as the CAPS-IV or CAPS-5. Furthermore, while the presence of physical injury was recorded, the precise timing of the injury relative to blood sampling could not be ascertained due to the realities of the combat setting, and this temporal ambiguity could significantly influence biomarker levels. The exclusive inclusion of male participants limits the generalizability of the findings to female veterans, though recruiting enough female combat veterans presents a practical challenge. The diagnostic algorithm was developed and validated on a specific sample of veterans seeking inpatient psychiatric care, and its accuracy requires further external validation in more diverse populations, including primary care settings and civilians with PTSD. Finally, the possible modulating effects of psychotropic medications on the HPA axis and inflammatory markers were not accounted for in the analysis.

## 5. Conclusions

Our study aimed to identify significant biological markers of impaired BBB permeability, HPA axis function, and neuroinflammation successfully developed a diagnostic algorithm for combat-related PTSD based on biomarkers of key pathophysiological pathways. The model, defined by an IL-6 level > 0.002438 pg/mL and a cortisol level < 199.8 nmol/L, achieved a correct classification rate of 78%. The use of these routinely available and cost-effective assays facilitates the potential integration of this algorithm into standard clinical workflows, demonstrating its promise for application in large patient populations pending further validation.

A second major finding was the identification of antibodies against neurospecific proteins as indicators of BBB compromise. Notably, anti-GFAP antibodies were elevated in all individuals exposed to combat trauma, irrespective of a subsequent PTSD diagnosis. This suggests that the traumatic event itself triggers an acute neuroinflammatory and autoimmune cascade against central nervous system tissue, a process that appears distinct from the development of chronic psychopathol-ogy.

Furthermore, our research established a direct association between the severity of combat-related psychological stress and the intensity of the neuroinflammatory response, independent of physical injury or pain. This finding implies that assessing specific neuroinflammatory markers could aid in the early identification of at-risk individuals, even before the full manifestation of PTSD, enabling timely preventative interventions for this vulnerable subgroup.

## Figures and Tables

**Table 1:** Data on the severity of stress and clinical data of PTSD and noPTSD groups. The analysis was performed using Kruskal–Wallis test. Significant *p*-values (<0.05) are highlighted in bold.

Parameters	noPTSD (*n* = 147)		PTSD (*n* = 434)		χ^2^	*p*
	Median	Q1; Q3	Median	Q1; Q3		
Duration of stress (being in a combat zone), weeks	26	4.3; 52	28	12; 37	0.002	0.96
The number of weeks after leaving the combat zone	6	4; 10	8	5; 13	3.8	0.051
The overall severity of PTSD symptoms	0.6	0.4; 1.4	1.4	0.8; 2.2	23.0	**<0.001**
Injury	11% (no)	89% (+)	13% (no)	87% (+)	0.602	0.438
Pain syndrome	10% (no)	90% (+)	16% (no)	84% (+)	2.544	0.111
**HADS**						
Depression	1	0; 3	3	1; 5	7.9	**0.005**
Anxiety	1	0; 3	3	1; 5	17.2	**<0.001**
**Likert scale**						
Depression	0	0; 0	1	1; 2	92.2	**<0.001**
Personality changes	0	0; 0	0	0; 1	25.3	**<0.001**
Avoidance	0	0; 0	1	0; 1	95.5	**<0.001**
Irritability	0	0; 0	1	0; 1	24.2	**<0.001**
Intrusions	0	0; 1	2	1; 3	145.8	**<0.001**
Sleep disturbances	1	0; 1	1	0; 1	0.08	0.77

**Table 2:** The results of quantitative assessment of blood biochemical parameters. The analysis was performed using Kruskal–Wallis test. Significant *p*-values (<0.05) are highlighted in bold.

Blood Biochemical Parameters	Control (*n* = 140)		noPTSD (*n* = 147)		PTSD (*n* = 434)		χ^2^	*p*
	Median	Q1; Q3	Median	Q1; Q3	Median	Q1; Q3		
Antibodies to GFAP	0.40	0.27; 0.58	0.67	0.54; 0.82	0.58	0.41; 0.73	60	**<0.001**
Antibodies to NSE	0.34	0.26; 0.42	0.57	0.38; 0.68	0.33	0.22; 0.46	28	**<0.001**
Cortisol	318	241; 427	209	126; 364	190	99; 307	68	**<0.001**
IL-6	0	0; 0	0	0; 0	1.25	0.41; 3.39	174	**<0.001**
IL-8	0	0; 8.25	7.9	0.0; 22.7	3.43	0.0; 16.5	24	**<0.001**

**Table 3:** Independent variables included in regression model, training sample.

	Significance (*p*)	Odds Ratio	95% CI for OR	
			Lower	Upper
IL-06	0.015	1.346	1.061	1.708
Cortisol	<0.0005	0.995	0.992	0.998
Constant	<0.0005	7.129		

## Data Availability

The data presented in this study are available on request from the corresponding author due to privacy and ethical reasons.

## Abbreviations

The following abbreviations are used in this manuscript:

BBB	Blood–brain barrier
GFAP	Glial fibrillary acid protein
HADS	Hospital Anxiety Depression Scale
HPA	Hypothalamic-pituitary axis
IL	Interleukin
NSE	Neuron specific enolase
PTSD	Directory of open access journals
